# Establishing a Gold Standard for Quantitative Menstrual Cycle Monitoring

**DOI:** 10.3390/medicina59091513

**Published:** 2023-08-23

**Authors:** Thomas Bouchard, Paul Yong, Patricia Doyle-Baker

**Affiliations:** 1 Department of Obstetrics & Gynecology, University of British Columbia, Vancouver, BC V6Z 2K8, Canada; pyong@cw.bc.ca; 2 Department of Family Medicine, University of Calgary, Calgary, AB T3H 0N9, Canada; 3 Faculty of Kinesiology, University of Calgary, Calgary, AB T2N 1N4, Canada; pdoyleba@ucalgary.ca

**Keywords:** ovulation, menstrual cycle, follicular-tracking ultrasound, polycystic ovarian syndrome, oligomenorrhea, follicle-stimulating hormone, FSH, estrone-3-glucuronide, E_1_3G, luteinizing hormone, LH, pregnanediol glucuronide, PDG

## Abstract

*Background and Objectives*: The Quantum Menstrual Health Monitoring Study will measure four key reproductive hormones in the urine (follicle-stimulating hormone, FSH; estrone-3-glucuronide, E_1_3G; luteinizing hormone, LH; and pregnanediol glucuronide, PDG) to characterize patterns that predict and confirm ovulation, referenced to serum hormones and the gold standard of the ultrasound day of ovulation in participants with regular cycles. These normal cycles will provide a reference for comparison to irregular cycles in subjects with polycystic ovarian syndrome (PCOS) and athletes. *Materials and Methods*: Participants will track their menstrual cycles for 3 months and be provided with an at-home urine hormone monitor (Mira monitor) to predict ovulation. The day of ovulation will be confirmed with serial ultrasounds completed in a community clinic. Urine results will be compared to serum hormone values. Other markers of menstrual health, such as bleeding patterns and temperature changes, will be determined using a customized app. Three groups will be recruited. Group 1 will include those with consistent regular cycle lengths (between 24–38 days), and will be compared to two groups with irregular cycle lengths (with increased cycle length variability and longer cycles). Group 2 will include those with polycystic ovarian syndrome (PCOS) with irregular cycles and Group 3 will include individuals participating in high levels of exercise with irregular cycles. *Hypothesis*: The Mira monitor quantitative urine hormone pattern will accurately correlate with serum hormonal levels and will predict (with LH) and confirm (with PDG) the ultrasound day of ovulation in those with regular cycles as well as those with irregular cycles. *Rationale*: Once the ultrasound validation is complete, tools like the Mira monitor with a customized app may become a new standard for at-home and remote clinical monitoring of the menstrual cycle without having to use labor-intensive follicular-tracking ultrasound or follow serum hormone changes. *Conclusions*: Precision monitoring of the menstrual cycle is expected to impact individuals who want to increase their menstrual health literacy and guide decisions about fertility.

## 1. Introduction

### 1.1. Background

**Figure 1:**
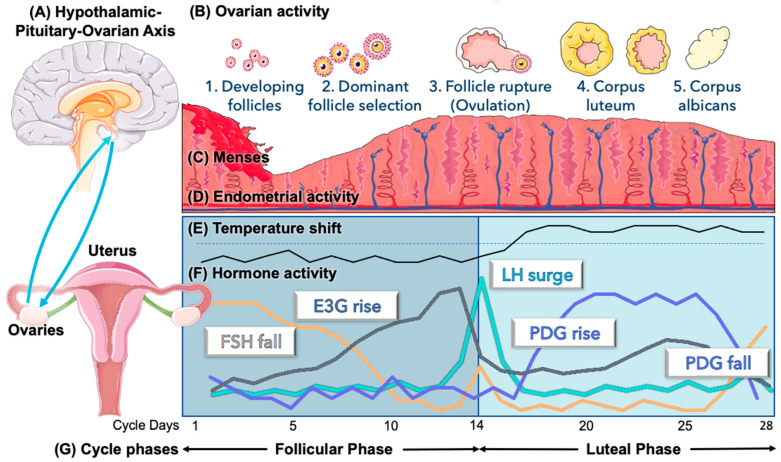
Ovarian–uterine (menstrual) cycle. (**A**) The hypothalamic–pituitary–ovarian (HPO) axis involves hormonal feedback loops between the ovaries, the hypothalamus, and the pituitary, which regulate the reproductive hormones and ovulation (**B**). A cycle begins with menses (**C**), the shedding of the endometrial lining (**D**), while follicle development is stimulated by FSH and estrogen (E_1_3G) to select a dominant follicle, which ruptures to release an egg under the stimulation of the LH surge (**F**). Progesterone rises after ovulation, which causes a basal body temperature shift (**E**) and supports the lining of the uterus (endometrium, (**D**)) to facilitate implantation of an embryo. If no embryo implants, progesterone falls (**F**), leading to menses (**C**). The follicular phase is from menses until ovulation, and the luteal phase is from ovulation until the end of the cycle (**G**). Adapted by T. Bouchard with permission from SERVIER Medical Art (smart.servier.com, accessed on 1 October 2022).

The menstrual cycle has been described as the “fifth vital sign” by professional associations [1], menstrual cycle researchers [2], and popular literature [3], reflecting a complex interaction between the hypothalamus, pituitary, and ovaries [4] (Figure 1). Observing this “vital sign” in a regular cycle can provide reassurance about the homeostasis of the reproductive system [2,4], while self-measured menstrual cycle changes could help to identify pathological states. Long and irregular menstrual cycles with anovulation are often reflective of underlying polycystic ovarian syndrome (PCOS) [5], which affects approximately 10% of reproductive-aged women [6]. Similar cycle disturbances are also present in athletes [7]. Other physiologic variations like the postpartum period [8] and perimenopause [9] have also been followed with menstrual cycle tracking [8,10]. There is, however, a significant gap in general knowledge of the menstrual cycle [11], and improving menstrual literacy [11,12] could be helpful for the common problem of infertility in North America [13], where the substantial need for care [14] may include both specialized care [15] and primary care approaches to infertility [16]. A simple primary care approach could include educating couples on the timing of intercourse with a personalized, at-home evaluation of urinary hormones [17]. Menstrual cycle hormones are usually evaluated with serum measurement of the key reproductive hormones shown in Figure 1; however, the complex dynamics of these hormones are inter-related on a day-to-day basis, so a single serum value in the cycle is less valuable than daily variations, which are more amenable to pattern recognition using urine hormones measured at home.

### 1.2. Irregular Cycles

PCOS is known to cause long and irregular menstrual cycles, related to an underlying ovulatory dysfunction [5,18]. Treating conditions like PCOS [19] can substantially improve couples’ chances of conceiving. PCOS is known to be associated with the development of endometrial cancer in diverse populations of women [20,21,22], likely driven by unopposed estrogen [23]. If women can identify this unopposed estrogen early on by monitoring their menstrual health, they may be able to address this pathology earlier to reduce the burden of this effect on endometrial cancer risk.

It seems obvious that the menstrual cycle may affect athletes’ performance, yet research efforts are needed to document how it does so to enable athletes and coaches to use the knowledge in training and performance plans [24]. It may also shed light on variability in the menstrual cycle. The menstrual cycle is not just perceived to influence the female athletes who experience it, but it also has excluded them from being used in scientific studies because it produced “white noise” in the results [7]. There is the possibility that we may improve aspects of physiological and psychological performance by understanding and monitoring athletes’ menstrual patterns, yet the quality of the literature regarding physiological effects has been criticized for inappropriate verification of the menstrual cycle phase [25]. In both circumstances, of PCOS and athletic menstrual cycle disturbances, menstrual cycle monitoring could serve as an “early warning system” to identify cycle disturbances.

### 1.3. Menstrual Cycle Monitoring

Digital health and personalized medicine are advancing at an unprecedented pace. There are now over one thousand smartphone apps to evaluate the menstrual cycle [26], but multiple studies in Canada and elsewhere have demonstrated that most of these apps are inaccurate [26,27,28]. Many people are searching for a more in-depth knowledge of the menstrual cycle [11], which fuels the high demand for these smartphone Apps, yet most of the apps available are unlikely to significantly improve users’ menstrual health literacy [29,30]. A second challenge with menstrual cycle apps is a lack of security and privacy, with some major apps having had data breaches for sharing users’ data [31], demonstrating the importance of ensuring these technological tools align with users’ best interests [32,33].

**Figure 2:**
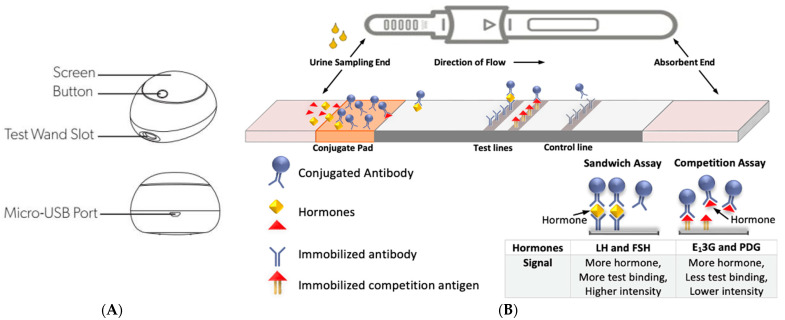
**Figure 2**. (**A**)—Mira Analyzer diagram from manufacturer (used with permission). (**B**)—Mira test stick lateral flow assay (**A**), based on a double antibody fluorescent labeling technique. LH and FSH are based on a sandwich assay and E_1_3G and PDG are based on a competition assay (**B**). For the sandwich assay, anti-LH or anti-FSH capture antibodies bind to LH and FSH in the urine and are bound to immobilized anti-LH and anti-FSH in the detection zone (as more hormone is present, there is a greater detection and stronger line intensity to be detected by the signal receiver). For the competition assay, E_1_3G and PDG in the urine compete with an immobilized binding antigen of these hormones, so if there is more hormone present, there is less binding and therefore a lower line intensity to be detected by the receiver (**A**). Adapted by T. Bouchard with permission from Radetech www.lateralflows.com/lateral-flow-assays/ accessed on 1 March 2023.

In addition to menstrual cycle apps, personalized menstrual health information can also be evaluated at home with urinary hormonal measurement. There are now quantitative fertility monitors, which give values of menstrual cycle hormones along with predictive algorithms provided in accompanying smartphone apps [34]. One of these devices, the Mira fertility monitor (Figure 2), measures follicle-stimulating hormone (FSH), estrone-3-glucuronide (E_1_3G), luteinizing hormone (LH), and pregnanediol glucuronide (PDG) in the urine [35].

### 1.4. Correlation with Menstrual Parameters, Serum, and Ultrasound

While there have been unpublished data demonstrating internal validity by the Mira monitor manufacturers, there have not been any formal studies comparing it to the gold standard of the estimated day of ovulation on ultrasound, and serum correlations need to be externally validated. These results should also be contextualized with regard to ovarian reserve with serum anti-Müllerian hormone (AMH) levels [36]. Besides hormonal variations, other key parameters of the menstrual cycle include bleeding patterns and vital sign changes. Bleeding patterns during menses and throughout the cycle are a barometer of menstrual health, and there are multiple bleeding scores to monitor these changes [37], but it is important to use a score that has been validated against a physical measurement of fluid loss like the Mansfield–Voda–Jorgensen Menstrual Bleeding Scale [38].

In addition, temperature changes in the menstrual cycle (i.e., the temperature shift after ovulation, see Figure 1E) have been known for many years and have been used to guide fertility assessment [39,40]. It should be emphasized that these menstrual cycle biomarkers require correlation with the gold standard of serum hormonal measurements and serial endovaginal ultrasound to track follicular development and ovulation. Previous studies have shown that the ultrasound-based day of ovulation is highly correlated with urinary hormones [41,42,43,44]. Our primary objective in this protocol will be to establish this correlation specifically for the Mira monitor.

## 2. Materials and Methods

### 2.1. Objective and Hypothesis

Our objective is to characterize quantitative hormones in the urine using the Mira monitor and validate these in reference to serum hormonal measurements and the gold standard of the ultrasound day of ovulation in participants with normal (regular) menstrual cycles. This will also help to establish external validity for the Mira monitor for use in women with regular cycles, rather than relying on industry-based internal data. We will then compare those with regular cycles to oligomenorrheic women with polycystic ovarian syndrome (PCOS) and oligomenorrheic athletes.

We hypothesize that the Mira monitor’s detected urine hormone pattern will accurately correlate with serum hormonal levels and will predict (LH) and confirm (PDG) the ultrasound day of ovulation in those with regular cycles as well as those with oligomenorrheic cycles.

### 2.2. Design

A prospective cohort with a longitudinal follow-up of participants with regular menstrual cycles and irregular cycles will be established in order to track urinary hormones with serum hormones and confirm ovulation with ultrasound.

### 2.3. Inclusion and Exclusion Criteria

Purposive sampling will be carried out to ensure an ethnically diverse sample reflective of the Canadian population. Table 1 and Table 2 outline the inclusion and exclusion criteria. These participants will be recruited through primary care clinics and university social media advertising along with snowball sampling in the community. Irregular cycles in PCOS will be identified based on historical cycle lengths and variability as well as one other Rotterdam criterion (Table 2). Irregular cycles in athletes will also be identified based on historical cycle lengths and variability, specifically when an athlete has recognized less frequent cycles with their training activities. Athletes will be recruited from known sports research groups locally.

### 2.4. Recruitment and Sample Size Considerations

Recruitment will occur through the University of Calgary social media and research sites. We anticipate that a snowball sampling effect will also occur. Interested participants will be contacted by a research assistant to complete an online survey to ensure they meet the inclusion criteria (Table 1 and Table 2).

Previous ultrasound studies validating urine hormone monitor measurements have targeted 50 participants over 3 cycles, for a total of 150 menstrual cycles [41]. With 150 cycles for analysis, the study would be adequately powered to detect differences of 0.5 days in the estimated day of ovulation, cycle length, and follicular/luteal phase lengths. This calculation was done with G*Power 3.1, with an effect size of 0.2, alpha 0.05, and power of 80%. This calculation is valid for correlation statistics with an a priori power analysis with one tail. Oversampling up to 60 participants may be required to ensure that we have 150 cycles for analysis. The same sample size will be used for recruitment of the PCOS and athlete groups. We know that the participant burden is high, and therefore, we are prepared to continue to recruit until we reach sample size.

### 2.5. Demographic and Clinical Surveys

The inclusion survey and consent will be completed on REDCap (Research Electronic Data Capture, www.project-redcap.org, accessed on 15 September 2022). If they meet the criteria, participants will review the Patient Information and Consent Form. After consenting via digital signature, they will be provided a link to enter demographic information within REDCap.

In addition to demographic data, a medical history questionnaire will be completed by each eligible participant directly in REDCap. The confidential (non-identifying) data is housed on the REDCap platform for the University of Calgary. Only investigators in this study will have access to the study data on REDCap.

**Figure 3:**
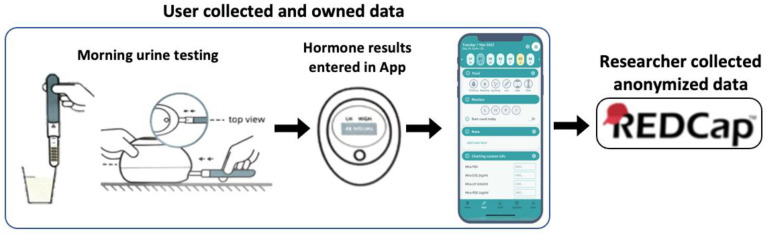
Data collection process for the study. Mira monitor urine results and other menstrual cycle observations are recorded in the Read Your Body (RYB) App. Users collect, control, and own the data they collect. RYB data is stored locally. Participants can then voluntarily send their anonymous data to a REDCap research database. Component figures used with permission.

Hormonal data from the Mira monitor will be entered directly into the Read Your Body App, developed by a non-profit women’s health initiative in the United Kingdom [46], and then sent directly to REDCap (see Figure 3). All data collection and analysis will follow provincial [47,48] and federal [49] privacy legislation. No biospecimens will be saved as part of this study (specimens are all home-based urine sampling with disposable test sticks).

### 2.6. Mira Monitor Testing Protocol

Participants will be given a Mira fertility monitor and enough test wands for three menstrual cycles. The Read Your Body (RYB) App (Figure 3) will need to be downloaded onto participants’ smartphones, and the participants will set up a personal accounts which the investigators will not have access to. The app developers have no way of accessing personally identifying details, nor menstrual cycle data, from the RYB app. Users of the app will have 3–4 months of free use of the App during the study. Email addresses and any other personally identifying details will not be collected by the app team nor by the research team through the app. Over the three menstrual cycles, participants will test daily first-morning urine (using an initial stream catch in a clean cup) with the Mira monitor for menstrual cycle hormones (FSH, E_1_3G, LH, and PDG) as well as take their temperature with a basal body thermometer. Data from the Mira monitor, menstrual cycle observations and bleeding patterns will be entered manually into the Read Your Body App. Bleeding patterns will be recorded with the bleeding score integrated in the app (see Section 2.7), and other observations (intercourse patterns, mucus secretions, open text observations) are also optionally available for manual entry in the app. The app provides cycle summaries and visual graphs of cycle changes to help improve menstrual health literacy for the individual user.

### 2.7. Menstrual Bleeding Assessment

The bleeding score for this study will be based on the Mansfield–Voda–Jorgensen (MVJ) Menstrual Bleeding Scale [38]. This is a 1–6 score, to which we will add a category 0 for “no bleeding.” This score will be built into the RYB App with explanations for each score available for participants’ reference:

(0) No bleeding.

(1) Spotting, a drop or two of blood, not even requiring sanitary protection though you may prefer to use some.

(2) Very light bleeding (you would need to change the least absorbent tampon or pad one or two times per day, though you may prefer to change more frequently).

(3) Light bleeding (you would need to change a low or regular absorbency tampon or pad two or three times per day, though you may prefer to change more frequently).

(4) Moderate bleeding (you would need to change a regular absorbency tampon or pad every 3 to 4 h, though you may prefer to change more frequently).

(5) Heavy bleeding (you would need to change a high absorbency tampon or pad every 3 to 4 h, though you may prefer to change more frequently).

(6) Very heavy bleeding or gushing (protection hardly works at all; you would need to change the highest absorbency tampon or pad every hour or two).

### 2.8. Serum Hormone Measurement

Serum hormones (FSH, estrogen, LH, progesterone) will be measured in the follicular phase, around the time of ovulation, and in the luteal phase. Serum AMH will be collected once for each participant. These tests are collected in lithium heparin tubes and analyzed on an E801 Cobas Roche analyzer. For progesterone and estradiol, the serum assay will use the competition principle, and for FSH and LH, the serum assay will use the sandwich principle (similar to the urine tests described in Figure 3).

### 2.9. Ultrasound Protocol

Follicular tracking ultrasounds will be carried out on a single machine (Canon, Xario 200G [50], regularly serviced by Canon technicians) in one clinic in Calgary with approximately 6–8 ultrasounds per cycle, for a total of 18–24 ultrasounds over the course of the three cycles of study. The endovaginal ultrasound protocol will be supervised by a radiologist and has been developed based on previous follicular-tracking ultrasound studies [51,52]. Ultrasound scans will be carried out by trained ultrasound technologists with endovaginal follicle-tracking experience. Processing and high-level disinfection of the ultrasound probes will be in compliance with the protocols recommended by the ultrasound probe manufacturer (Canon) and the College of Physicians and Surgeons as used by radiology clinics. A radiologist will be involved in identifying pitfalls and updating protocols throughout the project. The ultrasound machine and hard copies of ultrasound probe-processing tracking will be kept in a locked office. The ultrasound scans are kept locally on the ultrasound hard drive and will not be uploaded to any other system.

## 3. Results (Planned)

### 3.1. Data Analysis

Datasets will be analyzed using SPSS version 29 (IBM, Chicago, IL, USA) and charts prepared using SPSS or Excel (Microsoft, Redmond, WA, USA). Descriptive statistics will be used to determine the means, standard deviations (SD), and 95% confidence intervals (CI) of the menstrual cycle parameters, i.e., cycle length, day of ovulation, length of the follicular phase and the luteal phase, menstrual bleeding scores, and duration. The ultrasound-defined day of ovulation (US-DO) will be considered day 0 as a reference for days leading up to and after ovulation. The main analysis in this sample (for which it is powered) will be to correlate (with Spearman’s correlation coefficients) the day of ovulation with peak levels of LH on the Mira monitor, as well as with the day of progesterone elevation, to confirm ovulation. Sensitivity and specificity analyses of each hormone in relation to the US-DO will be calculated.

Secondary exploratory data analyses (not considered in the power calculations) will include daily average hormone values from the Mira monitor referenced to day 0 (US-DO), average hormone values, 95% confidence individuals on days leading up to ovulation (days −5, −4, −3, −2, −1, etc.), and after ovulation (days +1, +2, +3, +4, +5 etc.) from cycle day 6 until the end of the cycle. Daily means, SDs, and CIs of hormone values across the study sample will be used to describe the variability in the population, as was done in the pilot study [34], and individual (personalized) hormone patterns will also be considered as we have presented in previous studies [51]. Day-to-day hormone variability will require complex modelling of hormonal patterns, which we have used in previous studies [53], using *R*-software (*R* Foundation for Statistical Computing, Vienna, Austria). The specific statistical methods involve creating best-fit distributions for wave patterns of the hormones using the mean square of residuals as the loss function, with the goal to minimize the loss function to improve the fit of the distribution based on the density of the hormone wave patterns in the population; this specific method has helped to identify distinct hormone patterns that may represent different physiologic mechanisms, but it must be emphasized that these are hypothesis-generating rather than primary endpoints of this proposal.

### 3.2. Pitfalls and Mitigation Strategies

Frequent endovaginal ultrasounds may not be acceptable for the duration of the three cycles of use being requested of participants. To mitigate this, we will use trained ultrasound technologists and a patient-centered approach to provide participants with an acceptable level of discomfort during the procedure. A research assistant will follow up to ensure that ultrasound schedule reminders are provided to avoid missing follicular scans. During recruitment, we will seek subjects who would feel comfortable with this frequency of ultrasounds. We will oversample up to 60 participants, as stated earlier, to ensure we can have 150 cycles for analysis to achieve the appropriate power for our study.

Correlating progesterone changes with temperature and ultrasound-confirmed ovulation will help to validate progesterone as a confirmation of ovulation. Some studies have identified inconsistencies in specific PDG levels in the confirmation of ovulation [54], while other studies with ultrasound-confirmed ovulation have been able to identify relevant PDG levels to confirm ovulation [55].

### 3.3. Anticipated Results

We expect that the Mira monitor will accurately and precisely identify and confirm the day of ovulation as shown on ultrasound. Bleeding patterns and menstrual cycle observations will provide a baseline pattern for normal healthy participants to compare to abnormal cycles. We may be able to further expand on previous work related to daily quantitative hormone patterns, for example, by modelling estrogen changes from follicular wave activity in the early follicular phase [56], demonstrating the diversity of LH profiles related to ovulation patterns [57], or showing luteal progesterone variability [52,58]. The results from this study will potentially provide nomograms for urinary hormone measurements and provide reference values for future studies evaluating menstrual health.

## 4. Discussion

### 4.1. Regulatory Processes

Health Canada provided Investigational Testing Authorization (ITA, Class II) in June 2020 (Application No. 305611) for our pilot study, and an updated ITA has been submitted for the present study (Application No. 360587). The Ethics Board on record will be the Conjoint Health Research Ethics Board (CHREB) at the University of Calgary. This study is registered under clinicaltrials.gov and the record has been released and made public (https://clinicaltrials.gov/study/NCT05936840, accessed on 7 July 2023).

### 4.2. Establishing a Gold Standard for Menstrual Cycle Monitoring

Previous well-planned studies like this one have provided valuable and relevant data for analysis for two decades after their original collection [51,52,55]. We will be building on previous menstrual health research and incorporating new technological and personalized tools (i.e., the Mira monitor, the RYB App) with a focus on validation, privacy, security, and analytic precision. We hope that these tools will empower women to keep track of their menstrual health and identify changes that might provide early warnings for abnormalities in their menstrual cycles. More complex tools like the Mira hormone monitor give women precision and accuracy in planning for conception or for identifying fertility in difficult circumstances (e.g., perimenopause and postpartum). Having other tools that reduce the participant burden such as a thermometer or other wearable temperature sensors validated for menstrual cycle prediction will help athletes and others who want to employ new wearable technologies to monitor menstrual health that are easily incorporated in daily routines. A reliable and private app like the Read Your Body App will provide users with ways to track their cycle accurately without worrying about data breaches.

Participants in the sample with regular cycles or oligomenorrhea will hopefully identify important insights with the urinary hormones and menstrual cycle monitoring to guide decisions about fertility, particularly in the context of PCOS and oligomenorrhea in athletes, since these are relatively hypofertile states. Moreover, knowledge of the menstrual cycle allows people with PCOS to manage the unopposed estrogen long-term (with appropriate treatments to regulate the cycle in PCOS) and thus decrease the overall risk of endometrial cancer. Athletes may want to have easy methods to predict their menstrual cycle patterns to plan training and performance accordingly. Although urine hormone measurements may not be practical for many busy athletes, if we are able to precisely correlate hormone findings with vital signs, wearable biosensor technology may help to identify their menstrual cycle changes.

### 4.3. Economic Benefit

The validation of the Mira monitor for tracking the health of the menstrual cycle could have significant economic benefits for individuals and the health system as a whole.

Follicular tracking ultrasound for the purposes of identifying a healthy ovulation pattern can be expensive. There are usually 3–6 ultrasounds as part of the protocol, and considering the time for an ultrasound technician to perform the scan and a radiologist to review the scan, the cost per scan is likely around CAD 50–80, which would be CAD 150–480 per cycle.

Serum hormone tests are approximately CAD 15 per assay for estrogen, progesterone, FSH, and LH. If this were done 2–3 times per cycle (e.g., early follicular, peri-ovulatory, mid-luteal), this would cost CAD 180 per cycle. By contrast, the Mira monitor has an upfront cost of CAD 250, with a monthly cost of CAD 25–60 per cycle for test sticks that are CAD 3–4 each. Since the Mira monitor can be used for several years, the monthly cost to use the monitor would be one-third of the cost of serum measurements (which would be fewer in number), and it is even more economical compared to follicular tracking ultrasound.

The other important consideration of this economic benefit is that the Mira monitor is used at home, with no cost for travel nor for in-person visits to a provider to obtain requisitions. Moreover, the Mira monitor provides data about day-to-day changes to show hormonal variability across the menstrual cycle, rather than providing only three days of values (in the case of serum measurements) or a single day identified as ovulatory (in the case of follicular tracking).

Validating this at-home urine monitoring tool for the sake of menstrual cycle literacy, and to track abnormalities or to help with fertility, would provide dramatic savings to the health care system as a whole. This is especially the case because if women are tracking on their own, they would be paying for the Mira monitor and test sticks out of pocket, sometimes covered by a health spending account with their private insurance. This would relieve the burden of having to see a physician, obtain requisitions, and go to the lab or diagnostic imaging facility to obtain the required tests. For 100 women tracking 12 menstrual cycles over 1 year, this would amount to saving CAD 216,000 in lab costs and CAD 378,000 in ultrasound costs for the health care system.

### 4.4. Future Directions

Precision monitoring of the menstrual cycle is expected to impact many women who want to increase their menstrual health literacy for health benefits. Once the ultrasound validation is complete, tools like the Mira monitor can be relied upon for tracking medical treatments for various conditions without having to include the gold standard of ultrasound in every study. In the future, dynamic daily urine hormone measurements may replace serum hormone evaluation of the menstrual cycle. These tools may also form part of adverse health outcomes monitoring, including gynecologic cancer prediction models.

In this context, with these validated menstrual cycle monitoring tools, future studies could evaluate other gynecologic states. In endometriosis, shorter and heavier menstrual cycles have been associated with an increased risk of endometriosis [59]. The mechanism of the shorter menstrual cycles is not known, but it may be hypothesized that luteal progesterone may play a role given that there is evidence of progesterone-receptor resistance in the endometrium of patients with endometriosis [60]. Being able to identify urinary progesterone correlates could help establish the mechanism of the short cycles in endometriosis. Menstrual cycle monitoring may be able to recognize abnormal bleeding patterns and other possible subtle variations in their menstrual cycle that may relate to endometriosis-associated subfertility.

Finally, women approaching their decline of fertility in perimenopause may have abnormal bleeding and menstrual cycle patterns. While these patterns may be part of the normal transition to menopause, for example with more frequent anovulation [61], it is also possible they reflect abnormal hormone patterns leading to endometrial hyperplasia. Urine hormone monitoring at this stage in life may contribute to predicting endometrial hyperplasia and identify those at risk of later development of endometrial cancer.

## Figures and Tables

**Table 1:** Inclusion and exclusion criteria for regular cycles.

Inclusion Criteria	Exclusion Criteria
Regularly menstruating participants aged 18–45 Consistent cycle lengths between 24–38 days Knowledge of previous 3 cycle lengths Negative pregnancy test at the beginning and at the end of each cycle Able to travel to clinic in Calgary for regular ultrasounds during the study period	Anovulation in the last 3 cycles Currently, or in the previous 3 months, on medications that are known to impair or stimulate ovulation (e.g., oral contraceptives, ovulation stimulants, etc.) Known conditions that impair ovulation or fertility: ▪ polycystic ovarian syndrome ▪ endometriosis ▪ pelvic inflammatory disease in the last year ▪ pituitary adenoma ▪ breastfeeding Previous surgeries impacting the menstrual cycle: ▪ hysterectomy ▪ bilateral oophorectomy Currently pregnant

**Table 2:** Inclusion and exclusion criteria for oligomenorrheic cycles.

Inclusion Criteria	Exclusion Criteria
Participants aged 18–45 With a clinical diagnosis of PCOS (Rotterdam criteria [45] to include oligomenorrheic cycles and one of either polycystic ovaries or signs of excess androgens) OR Oligomenorrheic athletes	Currently, or in the previous 3 months, on medications that are known to impair or stimulate ovulation (e.g., oral contraceptives, ovulation stimulants, etc.) Known conditions that impair ovulation or fertility: ▪ pelvic inflammatory disease in the last year ▪ pituitary adenoma ▪ breastfeeding Previous surgeries impacting the menstrual cycle: ▪ hysterectomy ▪ bilateral oophorectomy Currently pregnant

## Data Availability

In order to support open-science practices, the University of British Columbia and the University of Calgary are part of a network that supports the Borealis Dataverse (https://borealisdata.ca/dataverse, accessed on 15 December 2022), which stores anonymous statistics files from the study so that other researchers can verify and replicate our study’s statistical methods. In consenting to participate in the study, participants would also consent to anonymous, aggregated data from the study being stored in these open databases to support ongoing research. At no time will identifying information, like name, birth date, address, or location be included in such data. The extent of the risk of being re-identified through anonymous data is unlikely but not impossible.
